# Rethinking familial hypercholesterolemia screening: Quantifying missed diagnoses at the intersection of genetic testing and lipoprotein(a)

**DOI:** 10.1016/j.ajpc.2026.101682

**Published:** 2026-05-25

**Authors:** Emma Delaney, Victor Huerta, Lindsey R. Helvaty, Julie M. Clary, Benjamin M. Helm

**Affiliations:** a Indiana University School of Medicine, Dept. of Medical & Molecular Genetics, Indianapolis, IN, United States; b Indiana University Health, Indianapolis, IN, United States; c Indiana University School of Medicine, Dept. of Medicine, Division of Cardiology, Indianapolis, IN, United States

**Keywords:** Familial hypercholesterolemia, Lipoprotein(a), Precision medicine, Population screening, Genomic screening

## 1 Introduction

Familial hypercholesterolemia (FH) is a common monogenic disease affecting approximately 1 in 220 people globally [1]. Genetic diagnosis informs risk stratification, as having pathogenic/likely pathogenic variant(s) and ≥190mg/dL LDL-C levels result in a 22-fold increased risk of ASCVD [2]. While genetic testing has been recommended as the gold-standard for FH diagnosis, it remains underutilized in healthcare systems.

Historically, the diagnosis of FH was based on LDL-C levels, clinical/family history, and physical findings, informing the development of diagnostic criteria like the Dutch Lipid Clinic Network (DLCN), Simon Broome (SB), and the Make Early Diagnosis to Prevent Early Deaths (MEDPED). Few studies have evaluated their screening performance against genetically-confirmed FH cases. Additionally, elevated lipoprotein(a) [Lp(a)] contributes to LDL-C levels and can mimic FH phenotypes; however, since the DLCN, SB, and MEDPED criteria predate broader recognition of Lp(a), few studies have explored the impact of Lp(a) on their predictive performance [[3], [4], [5], [6]]. Given the lack of global standardization in evaluation for FH, this study aimed to assess the performance of the DLCN, SB, and MEDPED criteria against the gold-standard of genetic diagnosis and evaluated the impact of Lp(a) on screening performance.

## 2 Methods

### 2.1 Study design & participants

The study utilized a clinical registry of patients evaluated at the Indiana University Health Advanced Lipid Clinic between 2016 and May 2025. The Indiana University Institutional Review Board approved the registry (protocol #23,922). Please see supplemental methods for details of clinical practices, genetic diagnosis definitions, and screening performance metrics used.

The total cohort (n = 521) included probands evaluated for FH with completed genetic testing and available LDL-C levels. A subcohort of 382 participants also had available Lp(a) levels. Most patients were ≥18 years of age, but 21/382 (5.5%) were <18 years old. Patients with genetic FH identified through cascade testing or secondary findings from prior unrelated genetic testing were excluded from this study.

### 2.2 Statistical analysis

STARD (Standards for Reporting of Diagnostic Accuracy) guidelines for diagnostic accuracy studies was followed. Data were analyzed using Statistical Analysis System (SAS v9.4). We reported descriptive statistics for demographics, comorbidities, clinical characteristics, and family history. They were then stratified by FH diagnosis utilizing chi-squared tests (Χ^2^). Screening performance metrics were calculated for the clinical diagnostic criteria and reported as percentages. Each participant was assigned a DLCN score and FH classification for SB and MEDPED. Binary thresholds for the DLCN were used to indicate a “screen-positive” status for FH: DLCN ≥3, DLCN ≥4, DLCN ≥6, and DLCN >8. A possible or definite SB classification signified a “screen-positive” status as did a MEDPED classification of “FH”. We also assessed the impact of Lp(a) on screening performance using a stratified analysis of Lp(a) <75mg/dL versus Lp(a) ≥75mg/dL. Lp(a) ≥75mg/dL was selected due to its contribution to measured LDL-C levels, and by extension, impact on clinical diagnostic criteria classification. For complete details, please see the supplemental methods.

## 3 Results

### 3.1 Demographics and clinical characteristics stratified by genetic FH diagnosis

**Fig. 1:**
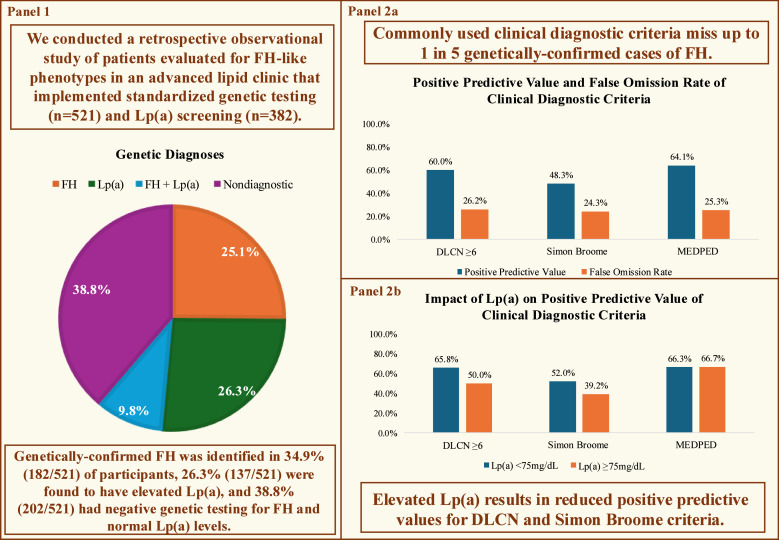
The false omission rate (FOR) exceeded 22% across all DLCN thresholds, with the highest value observed for DLCN >8 (31.6%). The FOR of SB was 24.3%, resulting in nearly 1 in 4 participants with genetically-confirmed FH being missed. Similarly, the FOR of MEDPED was 25.3%.

Demographics and clinical characteristics are summarized in Supplemental Table 1. A genetic diagnosis of FH was confirmed in 34.9% (182/521) of participants (Fig. 1, Panel 1). Demographic and clinical characteristics were stratified by genetic FH diagnosis (Supplemental Table 3). Participants with Lp(a) levels <75 mg/dL were more likely to have genetic FH (p = 0.017). In those with Lp(a) <75mg/dL, genetic FH was identified in 39.5% (n = 109/276).

### 3.2 Screening performance of clinical diagnostic criteria (Supplemental Table 4)

The PPV increased as the DLCN thresholds increased; the PPV for DLCN ≥3 was 37.0% [95% CI: 32%, 42%] while the PPV for DLCN >8 was 67.4% [95% CI: 64%, 73%]. The PPV for SB was 48.3% [95% CI: 42%, 55%] and 64.1% [95% CI: 56%, 72%] for MEDPED (Fig. 1, Panel 2a). However, the false omission rate (FOR) was approximately 25% for the clinical diagnostic criteria, indicating up to one in four cases of FH would be missed.

### 3.3 Screening performance stratified by Lp(a)

Participants were grouped into Lp(a) <75mg/dL (72.3%) and Lp(a) ≥75mg/dL (27.7%) [Supplemental Table 6]. The PPV decreased by at least 11.6% among participants with Lp(a) ≥75mg/dL compared to Lp(a) <75mg/dL across all DLCN thresholds and SB. There was no difference observed in the PPV for MEDPED when stratified by Lp(a). The FOR decreased by at least 11.2% across all DLCN thresholds, SB, and MEDPED criteria when accounting for elevated Lp(a) levels (≥75 mg/dL) compared with Lp(a) levels <75 mg/dL.

### 3.4 Sensitivity analyses

After excluding patients with DLCN scores ≤2, we found that key screening performance metrics did not change substantially overall and across Lp(a) levels. When excluding those <18 years, the FOR decreased slightly, but ranged ∼14–25% (mean FOR=18%). See supplemental materials for details.

## 4 Discussion

This is one of few studies to systematically assess the screening performance of clinical diagnostic criteria for FH in patients undergoing standardized genetic and Lp(a) testing. This work supports previously reported performance metrics for the DLCN, SB, and MEDPED criteria; our study extends this work by emphasizing the false omission rate of the clinical diagnostic criteria. Across DLCN thresholds the FOR was at least 22%. The FOR of SB and MEDPED was 24.3% and 25.3%, respectively. These findings suggest that even if a clinician was to define a lower threshold for ordering genetic testing, e.g., DLCN score ≥4, it would result in approximately 1 in 5 missed diagnoses in an enriched patient population such as our clinic. At the general population level, studies have shown a larger discrepancy between those who have genetic FH and those who meet clinical diagnostic criteria. Samadder et al. [7] reported only 30.8% of patients identified to have genetically-confirmed FH through whole exome sequencing met DLCN ≥3 criteria. Our results similarly support the need for new FH screening methods as overreliance upon current criteria may be hindering FH case-finding and missing treatment opportunities.

Lp(a) can clinically mimic FH, and our results show how elevated Lp(a) confounds the clinical diagnostic criteria and lowers the PPV. This finding underscores the critical need for routine Lp(a) testing for all patients evaluated for FH, as distinguishing patient diagnoses impacts clinical decision-making, treatment, and cascade testing in families.

### 4.1 Study limitations

Our sample is enriched with genetically-confirmed FH patients from a specialized lipid clinic and may not be generalizable to broader populations due to clinic sampling and referral biases. Our genetic testing practices in those with DLCN <3 are not as standardized but based on accounting for other variables, therefore, genetic diagnoses may be underestimated. We are unable to investigate the potential impact of polygenic causes of hypercholesterolemia since polygenic scores are not incorporated as a clinical standard. FH phenocopies like sitosterolemia and *APOE*-related hypercholesterolemia could be under-ascertained; however, given their rarity, the possible influence is likely low in this sample.

## 5 Conclusions

Our study demonstrated elevated Lp(a) influences screening for FH, as the PPV for the clinical diagnostic criteria decreased when Lp(a) was ≥75mg/dL. This potentially suggests many patients described as being clinically diagnosed with FH may have elevated Lp(a) instead. The findings of this study support that all patients with FH-like phenotypes should have genetic testing for FH and Lp(a) screening concurrently to optimize detection, specify risk and clinical management, and to ensure a complete and accurate evaluation.

## Ethical statement

This work has been carried out in accordance with The Code of Ethics of the World Medical Association and is in line with the Recommendations for the Conduct, Reporting, Editing and Publication of Scholarly Work in Medical Journals and aims for the inclusion of representative human populations. IRB approval was obtained through Indiana University (protocol #23,922).

## Funding

This research did not receive any specific grant from funding agencies in the public, commercial, or not-for-profit sectors.

## Use of AI and AI-assisted technologies statement

During the preparation of this work, the authors did not use AI or AI-assisted technologies.

## Author agreement

This work was approved by all authors.

## CRediT authorship contribution statement

**Emma Delaney:** Writing – original draft, Project administration, Methodology, Data curation, Conceptualization. **Victor Huerta:** Writing – review & editing, Conceptualization. **Lindsey R. Helvaty:** Writing – review & editing, Conceptualization. **Julie M. Clary:** Writing – review & editing, Conceptualization. **Benjamin M. Helm:** Writing – review & editing, Supervision, Project administration, Methodology, Formal analysis, Data curation, Conceptualization.

## Declaration of competing interest

We have no financial or competing interests related to this work.

## Appendix Supplementary materials

Image, application 1
