# Supplementary material for: Rethinking familial hypercholesterolemia screening: Quantifying missed diagnoses at the intersection of genetic testing and lipoprotein(a)
Source: Am J Prev Cardiol. 2026 May 25;29:101682. doi: 10.1016/j.ajpc.2026.101682 (PMC13329568; doi:10.1016/j.ajpc.2026.101682)
Supplement: Supplementary file 1 [file mmc1.docx]

“Rethinking Familial Hypercholesterolemia Screening: Quantifying Missed Diagnoses at the Intersection of Genetic Testing and Lipoprotein(a)”

Supplemental Materials

Table of Contents

[Supplemental Methods 3](#_Toc230254711)

[Supplemental Results 4](#_Toc230254712)

[Supplemental Figure 1 Determination of Cohort and Subcohort Sample Sizes 7](#_Toc230254713)

[Supplemental Table 1 Demographics and Clinical Characteristics of Study Sample 8](#_Toc230254714)

[Supplemental Figure 2 Screening Classifications of Clinical Diagnostic Criteria: Cohort-Wide and across Genetically-Confirmed Familial Hypercholesterolemia Status 10](#_Toc230254715)

[Supplemental Table 2 Screening Classifications of Clinical Diagnostic Criteria: Cohort-Wide and across Genetically-Confirmed Familial Hypercholesterolemia Status. 11](#_Toc230254716)

[Supplemental Table 3 Demographics and Clinical Characteristics: Cohort-wide and across Genetically-Confirmed Familial Hypercholesterolemia Status. 12](#_Toc230254717)

[Supplemental Figure 3 Screening Performance Metrics of Clinical Diagnostic Criteria 15](#_Toc230254718)

[Supplemental Table 4 Screening Performance Metrics of the Clinical Diagnostic Criteria compared to Gold-Standard Genetic Diagnosis of Familial Hypercholesterolemia. 16](#_Toc230254719)

[Supplemental Table 5 Screening Performance of the Dutch Lipis Clinic Network (DLCN) in Participants <18 years. 17](#_Toc230254720)

[Supplemental Figure 4 Impact of Lp(a) on Positive Predictive Value and False Omission Rate of Clinical Diagnostic Criteria 19](#_Toc230254721)

[Supplemental Table 6 Impact of Lp(a) on Screening Performance Metrics of the Clinical Diagnostic Criteria compared to Gold-Standard Genetic Diagnosis of Familial Hypercholesterolemia. 20](#_Toc230254722)

[Supplemental Table 7 Impact of Lp(a) on Screening Performance Metrics of the Clinical Diagnostic Criteria compared to Gold-Standard Genetic Diagnosis of Familial Hypercholesterolemia – Excluding Patients with DLCN Score <3. 22](#_Toc230254723)

[Supplemental Table 8 Basic Descriptive Statistics of Lp(a) Level Categories and Familial Hypercholesterolemia/Lp(a) Diagnoses. Note: The “Nondiagnostic” status was assigned to people that had negative genetic testing for familial hypercholesterolemia and normal Lp(a). 25](#_Toc230254724)

[Supplemental Figure 5 Condition-Based and Prediction-Based Metrics of Diagnostic Criteria 26](#_Toc230254725)

[Supplemental Figure 6 Screening Performance for Simon Broome and MEDPED 27](#_Toc230254726)

[Supplemental Figure 7 Impact of Lp(a) on Performance Metrics at DLCN Thresholds of ≥3, ≥4, ≥6, and >8 28](#_Toc230254727)

[Supplemental Figure 8 Impact of Lp(a) on Performance of Simon Broome and MEDPED Criteria 29](#_Toc230254728)

[References 30](#_Toc230254729)

# **Supplemental Methods**

**Clinical Practices**

Data were obtained through medical record review. Patients had multidisciplinary evaluations by cardiologists/lipidologists and genetic counselors. During patient appointments, clinical and family histories were reviewed, a three- to four-generation pedigree was created, patients were evaluated for physical findings of FH, and they were assigned clinical diagnostic criteria scores/classifications. Generally, patients with DLCN scores ≥3 were prioritized for genetic testing, including some with DLCN scores <3. Lp(a) levels were ordered for these patients as well at the time of genetic testing if they were unknown.

**Clinical Data/Variables of Interest**

Clinical and demographic data were extracted from medical records and maintained in the study database. Recorded variables included sex, race, ethnicity, comorbidities, LDL-C levels (mg/dL), Lp(a) levels (mg/dL), and family history. Physical findings specific to FH, like tendinous xanthomata and premature arcus cornealis, were also documented. Each participant was assigned a DLCN, SB, and MEDPED score/classification. Genetic testing results were recorded in the database, including the affected gene and zygosity for pathogenic or likely pathogenic variants.

**Familial Hypercholesterolemia Screening Methods**

Each participant was assigned a DLCN score and FH classification for SB and MEDPED [32]. Participants with a score of 3-5 were classified as having possible FH, 6-8 as probable FH, and >8 as definite FH using the DLCN criteria. For further analysis, patients were grouped into DLCN ≥3, DLCN ≥4, DLCN ≥6, and DLCN >8. Participants were classified as having no, possible, or definite FH using the SB criteria. For this study, participants were subsequently grouped into either the no FH classification or possible/definite Simon Broome classification. The MEDPED criteria classified participants as either having FH or not having FH based on total cholesterol levels, age, and family history.

**Genetic Diagnosis of Familial Hypercholesterolemia**

Genetic testing was ordered for patients from CAP (College of American Pathologists) accredited and CLIA (Clinical Laboratory Improvement Amendments) certified clinical diagnostic laboratories. Next generation sequencing including deletion/duplication analysis was performed for *APOB, LDLR, LDLRAP1,* and *PCSK9*.

Participants were assigned as either having genetic FH or no FH based on the genetic testing results. If their testing resulted in a pathogenic or likely pathogenic variant affecting the *APOB, LDLR, LDLRAP1,* or *PCSK9* gene(s), they were classified as having genetic FH. Variant interpretation followed the American College of Medical Genetics and Genomics (ACMG)/Association for Molecular Pathology (AMP) guidelines [8]. Those with negative/nondiagnostic genetic testing and normal Lp(a) were classified as having nondiagnostic evaluations, where the underlying etiology may be presumed multifactorial/polygenic hypercholesterolemia.

# **Supplemental Results**

**Genetic Testing Results**

Of those who had positive genetic testing, 80.2% were found to have a pathogenic or likely pathogenic variant affecting the *LDLR* gene. This was followed by 15.9% with an *APOB* pathogenic variant (all being the common p.Arg3527Gln variant). Three participants had pathogenic or likely pathogenic variants in the *PCSK9* gene, and three were homozygous or compound heterozygous for *LDLR* pathogenic or likely pathogenic variants. Last, one participant was found to have double heterozygous FH (*LDLR* and *APOB*).

**Prevalence of Genetically-Confirmed Familial Hypercholesterolemia according to DLCN, Simon Broome, and MEDPED Criteria**

We then assessed the prevalence of genetic FH across clinical diagnostic criteria used for screening prior to genetic testing (Figure 2). Most participants (75%) were described as unlikely (19.6%) or possibly (54.5%) having FH, with about 25% considered to have probable (16.5%) or definite (9.4%) FH by clinical criteria. Of participants with unlikely and possible DLCN classifications, 26.5% and 26.1% were later found to have genetic FH, respectively. Over half of those classified with probable FH had genetic FH (55.8%), and 67.4% of those with a definite FH classification according to the DLCN were found to have genetic FH.

Using the Simon Broome criteria as a screen prior to genetic testing, categorizations included no FH (56.2%), possible FH (39.2%), and definite FH (4.6%). Almost one-quarter of participants (24.3%) with the no FH classification were later determined to have genetically-confirmed FH, while 45.1% with a possible FH classification had genetically-confirmed FH. Only 75% of those with a definite classification had genetic FH.

With MEDPED classification, we found that 75.2% (n=388/516) of participants did not have FH, while 24.8% (n=128/516) had FH. However, of those classified with “No FH”, 25.3% had genetically-confirmed FH determined by genetic testing, and only 64.1% of those with a classification of “FH” had genetically-confirmed FH.

**Screening Performance of Clinical Diagnostic Criteria**

*Sensitivity, Specificity, False Positive, False Negative Rates, and Negative Predictive Value*

Performance metrics for the clinical diagnostic criteria are summarized in Table 3. When evaluating the performance metrics of the DLCN criteria, four binary thresholds were used to signify “screen-positive” status for FH: DLCN ≥3 (80.4%), DLCN ≥4 (56.1%), DLCN ≥6 (25.9%), and DLCN >8 (9.4%). Sensitivity decreased while specificity increased with higher DLCN thresholds. For example, the sensitivity of DLCN >8 was 18.1% [95% CI: 13%, 24%], compared with 85.2% [95% CI: 80%, 90%] for DLCN ≥3. In contrast, specificity was 95.3% [95% CI: 93%, 98%] for DLCN >8 and decreased to 22.1% [95% CI: 18%, 27%] for DLCN ≥3, indicating that lower thresholds result in increased false-positive screens as expected.

SB was evaluated by grouping those classified as no FH (292/520) versus possible or definite FH (228/520). The sensitivity for SB was 60.8% [95% CI: 54%, 68%], and the specificity was 65.2% [95% CI: 60%, 70%]. MEDPED demonstrated a sensitivity of 45.6% [95% CI: 38%, 53%] and a higher specificity of 86.3% [95% CI: 83%, 90%].

As expected, the false positive rate decreased with higher DLCN thresholds as DLCN ≥3 was 77.9%, while DLCN >8 was only 4.7%. The false negative rate increased with higher DLCN thresholds: DLCN ≥3 (14.8%), DLCN ≥4 (28.0%), DLCN ≥6 (55.5%) and DLCN >8 (81.9%). The false positive rate for SB was 34.8%, and the false negative rate was 39.2%. MEDPED had a false positive rate of 13.7% and a false negative rate of 54.4%.

Negative predictive values decreased with increasing DLCN thresholds; however, the NPV for DLCN ≥4 was the highest at 77.7% [95% CI: 72%, 83%]. The NPV for SB was 75.7% [95% CI: 71%, 81%] and 74.7% [95% CI: 70%, 79%] for MEDPED.

#
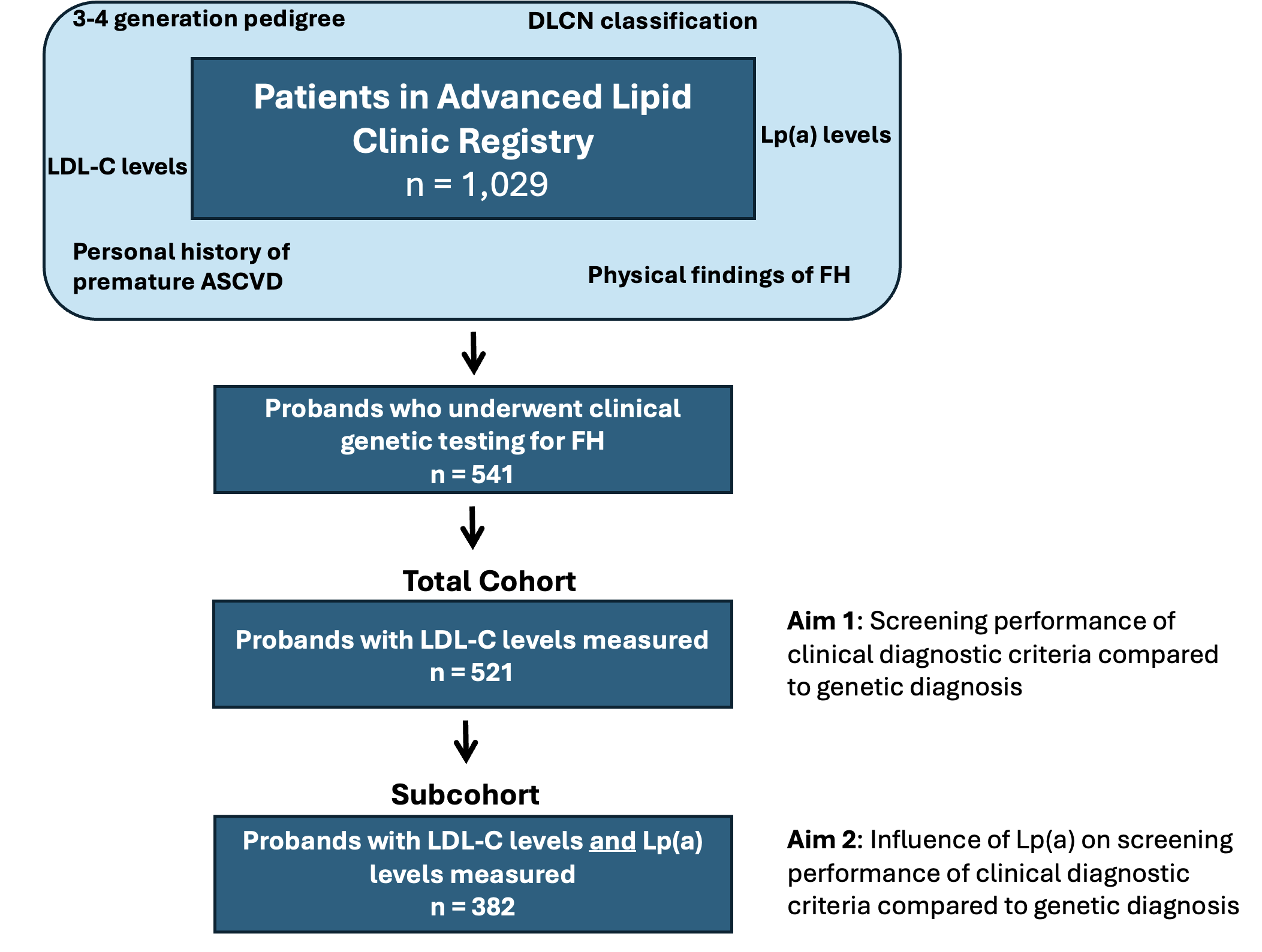
**Supplemental Figure 1** Determination of Cohort and Subcohort Sample Sizes

# **Supplemental Table 1** Demographics and Clinical Characteristics of Study Sample

| **Variable** | **Frequency (%) or Mean & SD**  **N=521** |
| --- | --- |
| **Age at Clinical Ascertainment** | N = 520  Mean = 42.7 years old  SD = 18.7 |
| **Sex** |  |
| Female | 313 (60.1%) |
| Male | 208 (39.9%) |
| **Race** |  |
| White | 413 (79.3%) |
| Black or African American | 70 (13.4%) |
| Asian | 25 (4.8%) |
| Native American | 2 (0.4%) |
| Native Hawaiian or Other Pacific Islander | 0 (0%) |
| Race Other | 12 (2.3%) |
| Race Not Reported/Unknown | 10 (1.9%) |
| **Ethnicity** |  |
| Non-Hispanic/Latino | 495 (95.0%) |
| Hispanic/Latino | 17 (3.3%) |
| Ashkenazi Jewish | 1 (0.2%) |
| Ethnicity Other | 6 (1.2%) |
| Ethnicity Not Reported/Unknown | 8 (1.5%) |
| **Comorbidities (at Baseline)** |  |
| Hyperlipidemia | 492 (94.4%) |
| Hypertension | 110 (21.2%) |
| Smoking History (current or former) | 83 (15.9%) |
| Anxiety | 69 (13.2%) |
| Obesity | 67 (12.9%) |
| Diabetes | 62 (11.9%) |
| Depression | 56 (10.8%) |
| Chronic Kidney Disease | 12 (2.3%) |
| **Treatment (at Baseline)** |  |
| Lipid-Lowering Therapy | 273 (52.4%) |
| **Physical Findings of Familial Hypercholesterolemia & Family History** | N=520 |
| Patient with premature coronary artery disease | 79 (15.2%) |
| Patient with premature cerebral or peripheral vascular disease | 11 (2.1%) |
| Patient with tendinous xanthomata | 24 (4.6%) |
| Patient with arcus cornealis (<age of 45) | 14 (2.7%) |
| First-degree relative(s) with known premature coronary and vascular disease, or first-degree relative(s) with known LDL-C >95^th^ percentile^1^ | 260/519 (50.1%) |
| First-degree relative(s) with tendinous xanthomata and/or arcus cornealis, or child(ren) with LDL-C >95^th^ percentile | 15 (2.9%) |
| **LDL-C Levels (mg/dL)** | N=521 |
| <130 | 18 (3.5%) |
| 130-159 | 25 (4.8%) |
| 160-189 | 64 (12.3%) |
| 190-220 | 114 (21.9%) |
| >220 | 300 (57.6%) |
| **Genetic Diagnoses (based on Genetic Testing Results)** | N=521 |
| Familial Hypercholesterolemia | 182 (34.9%) |
| Nondiagnostic | 339 (65.07%) |

Note: Family history and age at clinical ascertainment was unknown for one case resulting in sample size discrepancy.

# **Supplemental Figure 2** Screening Classifications of Clinical Diagnostic Criteria: Cohort-Wide and across Genetically-Confirmed Familial Hypercholesterolemia Status

# **Supplemental Table 2** Screening Classifications of Clinical Diagnostic Criteria: Cohort-Wide and across Genetically-Confirmed Familial Hypercholesterolemia Status.

| **Clinical Criteria/Scoring Classification** | **Overall Frequency (%)** | **Proportion without genetic diagnosis of FH (%)** | **Proportion with genetic diagnosis of FH (%)** |
| --- | --- | --- | --- |
| **Dutch Lipid Clinic Network Classifications** |  |  |  |
| Unlikely | 102/521 (19.6%) | 75/102 (73.5%) | 27/102 (26.5%) |
| Possible | 284/521 (54.5%) | 210/284 (73.9%) | 74/284 (26.1%) |
| Probable | 86/521 (16.5%) | 38/86 (44.2%) | 48/86 (55.8%) |
| Definite | 49/521 (9.4%) | 16/49 (32.7%) | 33/49 (67.4%) |
|  |  |  |  |
| **Simon Broome Classifications** |  |  |  |
| No/Unknown | 292/520 (56.2%) | 221/292 (75.7%) | 71/292 (24.3%) |
| Possible | 204/520 (39.2%) | 112/204 (54.9%) | 92/204 (45.1%) |
| Definite | 24/520 (4.6%) | 6/24 (25.0%) | 18/24 (75.0%) |
|  |  |  |  |
| **MEDPED Classifications** |  |  |  |
| No FH | 388/516 (75.2%) | 290/388 (74.7%) | 98/388 (25.3%) |
| FH | 128/516 (24.8%) | 46/128 (35.9%) | 82/ 128 (64.1%) |

Acronyms: FH = Familial Hypercholesterolemia, MEDPED = Make Early Diagnosis to Prevent Early Deaths

# **Supplemental Table 3** Demographics and Clinical Characteristics: Cohort-wide and across Genetically-Confirmed Familial Hypercholesterolemia Status.

| **Variable** | **Overall Frequency (%)** | **Proportion without genetic diagnosis of FH (%)** | **Proportion with genetic diagnosis of FH (%)** | **X^2^, Exact, or T-Test p-value** |
| --- | --- | --- | --- | --- |
| **Age at Clinical Ascertainment** | 520/521 (99.8%) | Mean = 47.3 years  (SD = 16.8) | Mean = 34.2 years (SD = 19.1) | **p-value <0.001** |
| **Sex** |  |  |  |  |
| Female | 313/521 (60.1%) | 211/313 (67.4%) | 102/313 (32.6%) | 0.17 |
| Male | 208/521 (39.9%) | 128/208 (61.5%) | 80/208 (38.5%) |  |
| **Race** |  |  |  |  |
| White | 413/521 (79.3%) | 264/413 (63.9%) | 149/413 (36.1%) | 0.28 |
| Black or African  American | 70/521 (13.4%) | 51/70 (72.9%) | 19/70 (27.1%) | 0.14 |
| Asian | 25/521 (4.8%) | 18/25 (72%) | 7/25 (28%) | 0.46 |
| Native American | 2/521 (0.4%) | 2/2 (100%) | 0 | 0.54 |
| Native Hawaiian or Other  Pacific Islander | 0/521 (0%) | 0 | 0 | N/A |
| Race Other | 12/521 (2.3%) | 7/12 (58.3%) | 5/12 (41.7%) | 0.76 |
| Race Not  Reported/Unknown | 10/521 (1.9%) | 6/10 (60%) | 4/10 (40%) | 0.75 |
| **Ethnicity** |  |  |  |  |
| Non-Hispanic/Latino | 495/521 (95.0%) | 322/495 (65.1%) | 173/495 (34.9%) |  |
| Hispanic/Latino | 17/521 (3.3%) | 10/17 (58.8%) | 7/17 (41.2%) | 0.58 |
| Ashkenazi Jewish | 1/521 (0.2%) | 1/1 (100%) | 0/1 (0%) | N/A |
| Ethnicity Other | 6/521 (1.2%) | 2/6 (33.3%) | 4/6 (66.7%) | 0.19 |
| Ethnicity Not  Reported/Unknown | 8/521 (1.5%) | 6/8 (75%) | 2/8 (25%) | 0.72 |
| **Comorbidities (at Baseline)** |  |  |  |  |
| Hyperlipidemia | 492/521 (94.4%) | 324/492 (65.9%) | 168/492 (34.2%) | 0.12 |
| Hypertension | 110/519 (21.2%) | 81/110 (73.6%) | 29/110 (26.4%) | **0.031** |
| Smoking History  (current or former) | 83/521 (15.9%) | 59/83 (71.1%) | 24/83 (28.9%) | 0.21 |
| Anxiety | 69/521 (13.2%) | 54/69 (78.3%) | 15/69 (21.7%) | **0.014** |
| Obesity | 67/521 (12.9%) | 45/67 (67.2%) | 22/67 (32.8%) | 0.70 |
| Diabetes | 62/521 (11.9%) | 50/62 (80.7%) | 12/62 (19.4%) | **0.006** |
| Depression | 56/521 (10.8%) | 46/56 (82.1%) | 10/56 (17.9%) | **0.005** |
| Chronic Kidney Disease | 12/521 (2.3%) | 9/12 (75%) | 3/12 (25%) | 0.56 |
| **Treatment (at Baseline)** |  |  |  |  |
| Lipid-Lowering Therapy | 273/521 (52.4%) | 180/273 (65.9%) | 93/273 (34.1%) | 0.66 |
| **Physical Findings of Familial Hypercholesterolemia & Family History** |  |  |  |  |
| Patient with premature  coronary artery disease | 79/520 (15.2%) | 52/79 (65.8%) | 27/79 (34.2%) | 0.90 |
| Patient with premature  cerebral or peripheral  vascular disease | 11/520 (2.1%) | 7/11 (63.6%) | 4/11 (36.4%) | 0.91 |
| Patient with tendinous  xanthomata | 24/520 (4.6%) | 7/24 (29.2%) | 17/24 (70.8%) | **<0.001** |
| Patient with arcus  cornealis (<age 45) | 14/520 (2.7%) | 5/14 (35.7%) | 9/14 (64.3%) | **0.019** |
| FDR with known  premature coronary and  vascular disease, or FDR  with known LDL-C  >95^th^ percentile | 260/519 (50.1%) | 153/260 (58.9%) | 107/260 (41.2%) | **0.003** |
| FDR with tendinous  xanthomata and/or arcus  cornealis, or child(ren)  with LDL-C >95^th^  percentile | 15/520 (2.9%) | 4/15 (26.7%) | 11/15 (73.3%) | **0.002** |
| **LDL-C Levels (mg/dL)** |  |  |  |  |
| <130 | 18/521 (3.5%) | 12/18 (66.7%) | 6/18 (33.3%) | **<0.001** |
| 130-159 | 25/521 (4.8%) | 20/25 (80.0%) | 5/25 (20.0%) |  |
| 160-189 | 64/521 (12.3%) | 51/64 (79.7%) | 13/64 (20.3%) |  |
| 190-220 | 114/521 (21.9%) | 97/114 (85.1%) | 17/114 (14.9%) |  |
| >220 | 300/521 (57.6%) | 159/300 (53.0%) | 141/300 (47.0%) |  |
| **Lp(a) Levels (mg/dL)** |  |  |  |  |
| <30 | 190/382 (49.7%) | 106/190 (55.8%) | 84/190 (44.2%) | **0.016** |
| 30-50 | 40/382 (10.5%) | 25/40 (62.5%) | 15/40 (37.5%) |  |
| 51-74 | 46/382 (12.0%) | 36/46 (78.3%) | 10/46 (21.7%) |  |
| 75-125 | 41/382 (10.7%) | 30/41 (73.2%) | 11/41 (26.8%) |  |
| 126 -180 | 36/382 (9.4%) | 27/36 (75.0%) | 9/36 (25.0%) |  |
| >180 | 29/382 (7.6%) | 21/29 (72.4%) | 8/29 (27.6%) |  |
| **Lp(a) Levels (binary) (mg/dL)** |  |  |  |  |
| <75 | 276/382 (72.3%) | 167/276 (60.5%) | 109/276 (39.5%) | **0.017** |
| ≥75 | 106/382 (27.7%) | 78/106 (73.6%) | 28/106 (26.4%) |  |

Acronym: FDR = first-degree relatives

# **Supplemental Figure 3** Screening Performance Metrics of Clinical Diagnostic Criteria

Acronyms: DLCN = Dutch Lipid Clinic Network, MEDPED = Make Early Diagnosis to Prevent Early Deaths

# **Supplemental Table 4** Screening Performance Metrics of the Clinical Diagnostic Criteria compared to Gold-Standard Genetic Diagnosis of Familial Hypercholesterolemia.

| **Screening Method** | **Sensitivity (%) (95% CI)** | **Specificity (%)**  **(95% CI)** | **FPR (%)**  **(95% CI)** | **FNR (%)**  **(95% CI)** | **PPV (%)**  **(95% CI)** | **NPV (%)**  **(95% CI)** | **FOR (%)**  **(95% CI)** | **FDR (%)**  **(95% CI)** |
| --- | --- | --- | --- | --- | --- | --- | --- | --- |
| **Dutch Lipid**  **Clinic Network Score**  **(N = 521)** | | | | | | | | |
| DLCN ≥3 | 85.2  (80, 90) | 22.1  (18, 27) | 77.9  (73, 82) | 14.8  (10, 20) | 37.0  (32, 42) | 73.5  (65, 82) | 26.5  (18, 35) | 63.0  (58, 68) |
| DLCN ≥4 | 72.0  (65, 79) | 52.5  (47, 58) | 47.5  (42, 53) | 28.0  (22, 35) | 44.9  (39, 51) | 77.7  (72, 83) | 22.3  (17, 28) | 55.1  (49, 61) |
| DLCN ≥6 | 44.5  (37, 52) | 84.1  (80, 88) | 15.9  (12, 20) | 55.5  (48, 63) | 60.0  (52, 68) | 73.8  (69, 78) | 26.2  (22, 31) | 40.0  (32, 48) |
| DLCN >8 | 18.1  (13, 24) | 95.3  (93, 98) | 4.7  (2, 7) | 81.9  (76, 87) | 67.4  (54, 80) | 68.4  (64, 73) | 31.6  (27, 36) | 32.7  (20, 46) |
| **Simon Broome**  **(N = 520)** | | | | | | | | |
| Possible/Definite vs. No | 60.8  (54, 68) | 65.2  (60, 70) | 34.8  (30, 40) | 39.2  (32, 46) | 48.3  (42, 55) | 75.7  (71, 81) | 24.3  (19, 29) | 51.8  (45, 58) |
| **MEDPED**  **(N = 516)** | | | | | | | | |
| FH vs. No FH | 45.6  (38, 53) | 86.3  (83, 90) | 13.7  (10, 17) | 54.4  (47, 62) | 64.1  (56, 72) | 74.7  (70, 79) | 25.3  (21, 30) | 35.9  (28, 44) |

Acronyms: CI = confidence interval, FDR = false discovery rate, FNR = false negative rate, FOR = false omission rate, FPR = false positive rate, MEDPED = Make Early Diagnosis to Prevent Early Deaths, NPV = negative predictive value, PPV = positive predictive value

Note: to reduce complexity of visual information displayed, confidence intervals were kept to zero decimal places.

# **Supplemental Table 5** Screening Performance of the Dutch Lipis Clinic Network (DLCN) in Participants <18 years.

| **DLCN Threshold** | **Sensitivity** | **Specificity** | **False-Positive Rate** | **Positive Predictive Value** | **Negative Predictive Value** | **False Omission Rate** |
| --- | --- | --- | --- | --- | --- | --- |
| DLCN≥3 (n=380/446) | 93.2% | 18.2% | 81.8% | 32.6% | 86.4% | 13.6% |
| DLCN≥4  (n=265/446) | 79.7% | 49.2% | 50.8% | 40.0% | 85.1% | 14.9% |
| DLCN≥6  (n=129/446) | 56.4% | 82.8% | 17.2% | 58.1% | 81.7% | 18.3% |
| DLCN>8  (n=49/446) | 24.8% | 94.9% | 5.1% | 67.4% | 74.8% | 25.2% |
|  | | | | | | **Average FOR** = 18% * |

*: Note that at an average FOR of 18%, this indicates that 1 in every 6 patients with FH would be missed. This shows that the younger patients <18 years do have an influence, though it may not be substantial.

- In the sub-cohort of patients with LDL-C and Lp(a) levels, 21/382 (5.5%) were age <18 years. These may not exert a strong influence on the overall findings from this study. A main theme is that we must often rely on LDL-C and personal/family history for screening, and we aim to highlight that as a limitation for FH case-finding. Otherwise, to investigate this further, we performed a narrow sensitivity analysis by excluding any patient <18 years in the overall sample and calculating screening performance.

This does seem to reflect the wider LDL-C distribution that occurs in younger patients:


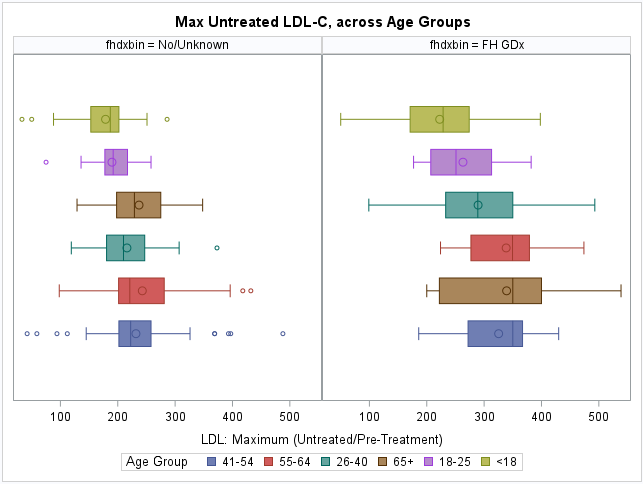


# **Supplemental Figure 4** Impact of Lp(a) on Positive Predictive Value and False Omission Rate of Clinical Diagnostic Criteria

# **Supplemental Table 6** Impact of Lp(a) on Screening Performance Metrics of the Clinical Diagnostic Criteria compared to Gold-Standard Genetic Diagnosis of Familial Hypercholesterolemia.

| **Screening Method** | **Sensitivity (%)**  **(95% CI)** | **Specificity (%)**  **(95% CI)** | **FPR (%)**  **(95% CI)** | **FNR (%)**  **(95% CI)** | | **PPV (%)**  **(95% CI)** | **NPV (%)**  **(95% CI)** | **FOR (%)**  **(95% CI)** | **FDR (%)**  **(95% CI)** |
| --- | --- | --- | --- | --- | --- | --- | --- | --- | --- |
| **Lp(a) < 75 mg/dL (N = 276)** | | | | | | | | | |
|  |  |  |  | |  |  |  |  |  |
| DLCN ≥3 | 88.1  (82, 94) | 22.2  (16, 28) | 77.8  (72, 84) | | 11.9  (6, 18) | 42.5  (36, 49) | 74.0  (62, 86) | 26.0  (14, 38) | 57.5  (51, 64) |
| DLCN ≥4 | 74.3  (66, 83) | 52.1  (45, 60) | 47.9  (40, 55) | | 25.7  (17, 34) | 50.3  (43, 58) | 75.7  (68, 84) | 24.4  (17, 32) | 49.7  (42, 57) |
| DLCN ≥6 | 45.9  (37, 55) | 84.4  (79, 90) | 15.6  (10, 21) | | 54.1  (45, 63) | 65.8  (55, 76) | 70.5  (64, 77) | 29.5  (23, 36) | 34.2  (24, 45) |
| DLCN >8 | 21.1  (13, 29) | 94.0  (90, 98) | 6.0  (2, 10) | | 78.9  (71, 87) | 69.7  (54, 85) | 64.6  (59, 71) | 35.4  (29, 41) | 30.3  (15, 46) |
|  |  |  |  | |  |  |  |  |  |
| Simon Broome (Possible/Definite) | 58.7  (49, 68) | 64.7  (57, 72) | 35.3  (28, 43) | | 41.3  (32, 51) | 52.0  (43, 61) | 70.6  (63, 78) | 29.4  (22, 37) | 48.0  (39, 57) |
|  |  |  |  | |  |  |  |  |  |
| MEDPED | 49.1  (40, 59) | 83.7  (78, 89) | 16.3  (11, 22) | | 50.9  (42, 60) | 66.3  (56, 77) | 71.7  (65, 78) | 28.4  (22, 35) | 33.8  (23, 44) |
|  |  |  |  | |  |  |  |  |  |
| **Lp(a) ≥75 mg/dL (N = 106)** | | | | | | | | | |
|  |  |  |  | |  |  |  |  |  |
| DLCN ≥3 | 96.4  (90, 100) | 14.1  (6, 22) | 85.9  (78, 94) | | 3.6  (0, 10) | 28.7  (20, 38) | 91.7  (76, 100) | 8.3  (0, 24) | 71.3  (62, 80) |
| DLCN ≥4 | 85.7  (73, 99) | 51.3  (40, 62) | 48.7  (38, 60) | | 14.3  (1, 27) | 38.7  (27, 51) | 90.9  (82, 99) | 9.1  (0, 18) | 61.3  (49, 73) |
| DLCN ≥6 | 57.1  (39, 75) | 79.5  (71, 88) | 20.5  (12, 29) | | 42.9  (25, 61) | 50.0  (33, 67) | 83.8  (75, 92) | 16.2  (8, 25) | 50.0  (33, 67) |
| DLCN >8 | 14.3  (1, 27) | 96.2  (92, 100) | 3.9  (0, 8) | | 85.7  (73, 99) | 57.1  (20, 94) | 75.8  (67, 84) | 24.2  (16, 33) | 42.9  (6, 80) |
|  |  |  |  | |  |  |  |  |  |
| Simon Broom  (Possible/Definite) | 71.4  (55, 88) | 60.3  (49, 71) | 39.7  (29, 51) | | 28.6  (12, 45) | 39.2  (26, 53) | 85.5  (76, 95) | 14.6  (5, 24) | 60.8  (47, 74) |
|  |  |  |  | |  |  |  |  |  |
| MEDPED | 57.1  (39, 75) | 89.6  (83, 96) | 10.4  (4, 17) | | 42.9  (25, 61) | 66.7  (48, 86) | 85.2  (77, 93) | 14.8  (7, 23) | 33.3  (14, 52) |

Acronyms: CI = confidence interval, FDR = false discovery rate, FNR = false negative rate, FOR = false omission rate, FPR = false positive rate, MEDPED = Make Early Diagnosis to Prevent Early Deaths, NPV = negative predictive value, PPV = positive predictive value

# **Supplemental Table 7** Impact of Lp(a) on Screening Performance Metrics of the Clinical Diagnostic Criteria compared to Gold-Standard Genetic Diagnosis of Familial Hypercholesterolemia – **Excluding Patients with DLCN Score <3.**

| **Screening Method** | **Sensitivity (%)**  **(95% CI)** | **Specificity (%)**  **(95% CI)** | **FPR (%)**  **(95% CI)** | **FNR (%)**  **(95% CI)** | | **PPV (%)**  **(95% CI)** | **NPV (%)**  **(95% CI)** | **FOR (%)**  **(95% CI)** | **FDR (%)**  **(95% CI)** |
| --- | --- | --- | --- | --- | --- | --- | --- | --- | --- |
| **Lp(a) < 75 mg/dL (N = 226)** | | | | | | | | | |
|  |  |  |  | |  |  |  |  |  |
| DLCN ≥4 | 84.4  (77, 92) | 38.4  (30, 47) | 61.6  (53, 70) | | 15.6  (8, 23) | 50.3  (43, 58) | 77.0  (67, 87) | 23.0  (13, 33) | 49.7  (42, 57) |
| DLCN ≥6 | 52.0  (42, 62) | 80.0  (73, 87) | 20.0  (13, 27) | | 48.0  (38, 58) | 65.8  (55, 76) | 69.3  (62, 77) | 30.7  (23, 38) | 34.2  (24, 45) |
| DLCN >8 | 24.0  (15, 33) | 92.3  (88, 97) | 7.7  (3, 12) | | 76.0  (67, 85) | 69.7  (54, 85) | 62.2  (55, 69) | 37.8  (31, 45) | 30.3  (15, 46) |
|  |  |  |  | |  |  |  |  |  |
| Simon Broome (Possible/Definite) | 64.6  (55, 74) | 59.2  (51, 68) | 40.8  (32, 49) | | 35.4  (26, 45) | 53.9  (45, 63) | 69.4  (61, 78) | 30.6  (22, 39) | 46.1  (37, 55) |
|  |  |  |  | |  |  |  |  |  |
| MEDPED | 55.8  (46, 66) | 82.2  (76, 89) | 17.8  (11, 24) | | 44.2  (34, 54) | 69.7  (59, 80) | 71.6  (64, 79) | 28.4  (21, 36) | 30.3  (20, 41) |
|  |  |  |  | |  |  |  |  |  |
| **Lp(a) ≥75 mg/dL (N = 94)** | | | | | | | | | |
|  |  |  |  | |  |  |  |  |  |
| DLCN ≥4 | 89.0  (77, 100) | 43.3  (31, 55) | 56.7  (45, 69) | | 11.0  (0, 23) | 38.7  (27, 51) | 90.6  (81, 100) | 9.4  (0, 19) | 61.3  (49, 73) |
| DLCN ≥6 | 59.3  (41, 78) | 76.2  (66, 86) | 23.8  (14, 34) | | 40.7  (22, 59) | 50.0  (33, 67) | 82.3  (73, 92) | 17.7  (8, 27) | 50.0  (33, 67) |
| DLCN >8 | 14.8  (1, 28) | 95.5  (91, 100) | 4.5  (0, 9) | | 85.2  (72, 99) | 57.1  (20, 94) | 73.6  (64, 83) | 26.4  (17, 37) | 42.9  (6, 80) |
|  |  |  |  | |  |  |  |  |  |
| Simon Broom  (Possible/Definite) | 70.4  (53, 88) | 56.7  (45, 69) | 43.3  (31, 55) | | 29.6  (12, 47) | 39.6  (26, 53) | 82.6  (72, 94) | 17.4  (6, 28) | 60.4  (47, 74) |
|  |  |  |  | |  |  |  |  |  |
| MEDPED | 59.3  (41, 78) | 87.9  (80, 96) | 12.1  (4, 20) | | 40.7  (22, 59) | 66.7  (48, 86) | 84.1  (75, 93) | 15.9  (7, 25) | 33.3  (14, 52) |

Acronyms: CI = confidence interval, FDR = false discovery rate, FNR = false negative rate, FOR = false omission rate, FPR = false positive rate, MEDPED = Make Early Diagnosis to Prevent Early Deaths, NPV = negative predictive value, PPV = positive predictive value

- We completed a narrow sensitivity analysis in the n=382 sub-sample to investigate the impact of excluding patients with DLCN scores <3 when assessing how Lp(a) influences screening performance of the clinical diagnostic criteria. Of note:
  - 62/382 (16.2%) of this sample had DLCN scores <3. Of these, the average age was 30.8 years (SD 18.9), with 21/62 (33.9%) being <18 years of age.
    - 8/62 (12.9%) had DLCN=0, 24 (38.7%) had DLCN=1, and 30 (48.4%) had DLCN=2. The average maximal LDL-C increased from 126 to 172 to 185 mg/dL across DLCN scores of 0, 1, and 2, respectively. This shows that DLCN scores of 1-2 were likely influenced by less striking LDL-C elevations (and those with DLCN=2 often having a family history criterion met as well).
    - Further, 71% (n=44) of these had maximal untreated LDL-C of 130-189 mg/dL, and 53.2% had Lp(a) <30 mg/dL while 46.8% had Lp(a) ≥30 mg/dL.
    - Only 2/62 (3.2%) had premature ASCVD, while 32 (51.6%) had a family history of a first-degree relative with premature ASCVD or high cholesterol.

This indicates that patients with DLCN scores <3 tended to have milder LDL-C elevations which would assign a DLCN score of 1, and patients may have additionally had DLCN score of 2 considering the family history. Notably, nearly half of these had Lp(a) elevations.

We then re-ran our key study outcomes measures including all screening performance metrics stratified by Lp(a) <75 and ≥75 mg/dL. This table is below. However, when focusing just on the impact of Lp(a) on key measures like PPV and FOR, the following was noted:

- For Lp(a)<75 mg/dL group:
  - DLCN4 threshold: PPV did not change after excluding the DLCN<3 patients.
    - FOR changed from 24.4% to 23.0% after excluding DLCN <3 patients.
  - DLCN6 threshold: PPV did not change after excluding the DLCN<3 patients.
    - FOR changed from 29.5% to 30.7% after excluding DLCN <3 patients.
  - DLCN8 threshold: PPV did not change after excluding the DLCN<3 patients.
    - FOR changed from 35.4% to 37.8% after excluding DLCN <3 patients
  - Simon Broome: PPV changed from 52% to 53.9% after excluding DLCN <3 patients.
    - FOR changed from 29.4% to 30.6% after excluding DLCN <3 patients.
  - MEDPED: PPV changed from 66.3% to 69.7% after excluding DLCN <3 patients
    - FOR did not change after excluding DLCN <3 patients.
- For the Lp(a)≥75 mg/dL group:
  - DLCN4 threshold: PPV did not change after excluding the DLCN<3 patients.
    - FOR changed from 9.1% to 9.4% after excluding DLCN <3 patients.
  - DLCN6 threshold: PPV did not change after excluding the DLCN<3 patients.
    - FOR changed from 16.2% to 17.7% after excluding DLCN <3 patients.
  - DLCN8 threshold: PPV did not change after excluding the DLCN<3 patients.
    - FOR changed from 24.2% to 26.4% after excluding DLCN <3 patients
  - Simon Broome: PPV changed from 39.2% to 39.6% after excluding DLCN <3 patients.
    - FOR changed from 14.6% to 17.4% after excluding DLCN <3 patients.
  - MEDPED: PPV did not change after excluding DLCN <3 patients
    - FOR changed from 14.8% to 15.9% after excluding DLCN <3 patients.

Altogether, this suggests that excluding patients with DLCN score <3 did not have a substantial impact on the screening performance metrics. The PPV either did not change or only minimally changed across both Lp(a) strata and the DLCN/SB/MEDPED criteria. The FOR also changed minimally (absolute difference of ~1-2%) across Lp(a) strata for the DLCN/SB/MEDPED criteria.

# **Supplemental Table 8** Basic Descriptive Statistics of Lp(a) Level Categories and Familial Hypercholesterolemia/Lp(a) Diagnoses. Note: The “Nondiagnostic” status was assigned to people that had negative genetic testing for familial hypercholesterolemia and normal Lp(a).

| **Variable** | **Frequency (%)** |
| --- | --- |
| **Lp(a) Levels (mg/dL)** | N=382 |
| <30 | 190 (49.7%) |
| 30-50 | 40 (10.5%) |
| 51-74 | 46 (12.0%) |
| 75-125 | 41 (10.7%) |
| 126 -180 | 36 (9.4%) |
| >180 | 29 (7.6%) |
| **Genetic Diagnoses** | N=521 |
| FH | 131 (25.1%) |
| Elevated Lp(a) | 137 (26.3%) |
| FH + Elevated Lp(a) | 51 (9.8%) |
| Nondiagnostic | 202 (38.8%) |

1. Lp(a) measurements were available for only 382 participants.
2. “Nondiagnostic” refers to patients who have had negative genetic testing and normal Lp(a) levels.

# **Supplemental Figure 5** Condition-Based and Prediction-Based Metrics of Diagnostic Criteria

# **Supplemental Figure 6** Screening Performance for Simon Broome and MEDPED

# **Supplemental Figure 7** Impact of Lp(a) on Performance Metrics at DLCN Thresholds of ≥3, ≥4, ≥6, and >8

# **Supplemental Figure 8** Impact of Lp(a) on Performance of Simon Broome and MEDPED Criteria

# **References**

1. Sturm, A.C., et al., *Clinical Genetic Testing for Familial Hypercholesterolemia: JACC Scientific Expert Panel.* J Am Coll Cardiol, 2018. **72**(6): p. 662-680.

2. Brown, M.S. and J.L. Goldstein, *Michael S. Brown, MD and Joseph L. Goldstein, MD. 1985 Nobel laureates in medicine.* J Investig Med, 1996. **44**(2): p. 14-23.

3. Humphries, S.E. and M. Futema, *Genetic Determinants of the Familial Hypercholesterolaemia Phenotype.* Ann Hum Genet, 2025: p. e12594.

4. Abul-Husn, N.S., et al., *Genetic identification of familial hypercholesterolemia within a single U.S. health care system.* Science, 2016. **354**(6319).

5. Khera, A.V., et al., *Diagnostic Yield and Clinical Utility of Sequencing Familial Hypercholesterolemia Genes in Patients With Severe Hypercholesterolemia.* J Am Coll Cardiol, 2016. **67**(22): p. 2578-89.

6. Cohen, J.C., et al., *Sequence variations in PCSK9, low LDL, and protection against coronary heart disease.* N Engl J Med, 2006. **354**(12): p. 1264-72.

7. Medeiros, A.M., et al., *Unraveling the genetic background of individuals with a clinical familial hypercholesterolemia phenotype.* J Lipid Res, 2024. **65**(2): p. 100490.

8. Zhang, Y., et al., *Association of Severe Hypercholesterolemia and Familial Hypercholesterolemia Genotype With Risk of Coronary Heart Disease.* Circulation, 2023. **147**(20): p. 1556-1559.

9. Nordestgaard, B.G., et al., *Familial hypercholesterolaemia is underdiagnosed and undertreated in the general population: guidance for clinicians to prevent coronary heart disease: consensus statement of the European Atherosclerosis Society.* Eur Heart J, 2013. **34**(45): p. 3478-90a.

10. Luirink, I.K., et al., *20-Year Follow-up of Statins in Children with Familial Hypercholesterolemia.* N Engl J Med, 2019. **381**(16): p. 1547-1556.

11. Ward, A., et al., *Underutilization of Guideline-Recommended Genetic Testing to Diagnose Familial Hypercholesterolemia in a Large, Real-world Cohort.* Journal of Clinical Lipidology, 2023. **17**(4): p. e31-e32.

12. Watts, G.F., et al., *Integrated guidance on the care of familial hypercholesterolaemia from the International FH Foundation.* Int J Cardiol, 2014. **171**(3): p. 309-25.

13. Humphries, S.E., et al., *What is the clinical utility of DNA testing in patients with familial hypercholesterolaemia?* Curr Opin Lipidol, 2008. **19**(4): p. 362-8.

14. Tricou, E.P., et al., *Genetic Testing for Familial Hypercholesterolemia in Clinical Practice.* Curr Atheroscler Rep, 2023. **25**(5): p. 197-208.

15. Defesche, J.C., et al., *Advanced method for the identification of patients with inherited hypercholesterolemia.* Semin Vasc Med, 2004. **4**(1): p. 59-65.

16. *Risk of fatal coronary heart disease in familial hypercholesterolaemia. Scientific Steering Committee on behalf of the Simon Broome Register Group.* Bmj, 1991. **303**(6807): p. 893-6.

17. Chan, D.C., et al., *A Comparative Analysis of Phenotypic Predictors of Mutations in Familial Hypercholesterolemia.* J Clin Endocrinol Metab, 2018. **103**(4): p. 1704-1714.

18. Ahmad, Z.S., et al., *US physician practices for diagnosing familial hypercholesterolemia: data from the CASCADE-FH registry.* J Clin Lipidol, 2016. **10**(5): p. 1223-9.

19. Williams, R.R., et al., *Diagnosing heterozygous familial hypercholesterolemia using new practical criteria validated by molecular genetics.* Am J Cardiol, 1993. **72**(2): p. 171-6.

20. Hendricks-Sturrup, R.M. and C.Y. Lu, *Understanding Implementation Challenges to Genetic Testing for Familial Hypercholesterolemia in the United States.* J Pers Med, 2019. **9**(1).

21. Hendricks-Sturrup, R.M., et al., *Barriers and Facilitators to Genetic Testing for Familial Hypercholesterolemia in the United States: A Review.* J Pers Med, 2019. **9**(3).

22. Fleming, J.K., et al., *A strategy to increase identification of patients with Familial Hypercholesterolemia: Application of the Simon Broome lipid criteria in a large-scale retrospective analysis.* Am J Prev Cardiol, 2025. **21**: p. 100930.

23. Casula, M., et al., *Evaluation of the performance of Dutch Lipid Clinic Network score in an Italian FH population: The LIPIGEN study.* Atherosclerosis, 2018. **277**: p. 413-418.

24. Miserez, A.R., F.J. Martin, and D. Spirk, *DIAgnosis and Management Of familial hypercholesterolemia in a Nationwide Design (DIAMOND-FH): Prevalence in Switzerland, clinical characteristics and the diagnostic value of clinical scores.* Atherosclerosis, 2018. **277**: p. 282-288.

25. Ramsis, M., et al., *Lipoprotein(a) Testing Trends in the United States 2015-2024: An Analysis of 300 Million Individuals.* JACC Adv, 2025. **4**(11 Pt 1): p. 102205.

26. Trinder, M., et al., *Ascertainment Bias in the Association Between Elevated Lipoprotein(a) and Familial Hypercholesterolemia.* J Am Coll Cardiol, 2020. **75**(21): p. 2682-2693.

27. Langsted, A., et al., *High lipoprotein(a) as a possible cause of clinical familial hypercholesterolaemia: a prospective cohort study.* Lancet Diabetes Endocrinol, 2016. **4**(7): p. 577-87.

28. Ellis, K.L., et al., *Value of Measuring Lipoprotein(a) During Cascade Testing for Familial Hypercholesterolemia.* J Am Coll Cardiol, 2019. **73**(9): p. 1029-1039.

29. Olmastroni, E., et al., *Lipoprotein(a) Genotype Influences the Clinical Diagnosis of Familial Hypercholesterolemia.* J Am Heart Assoc, 2023. **12**(10): p. e029223.

30. Harris, P.A., et al., *The REDCap consortium: Building an international community of software platform partners.* J Biomed Inform, 2019. **95**: p. 103208.

31. Harris, P.A., et al., *Research electronic data capture (REDCap)--a metadata-driven methodology and workflow process for providing translational research informatics support.* J Biomed Inform, 2009. **42**(2): p. 377-81.

32. Lozano, P., Henrikson, N. B., Dunn, J., et al, *Lipid Screening in Childhood and Adolescence for Detection of Familial Hypercholesterolemia: A Systematic Evidence Review for the U.S. Preventive Services Task Force [Internet]*. 2016, Agency for Healthcare Research and Quality (US): Rockville, MD.

33. Richards, S., et al., *Standards and guidelines for the interpretation of sequence variants: a joint consensus recommendation of the American College of Medical Genetics and Genomics and the Association for Molecular Pathology.* Genet Med, 2015. **17**(5): p. 405-24.

34. Bossuyt, P.M., et al., *STARD 2015: An Updated List of Essential Items for Reporting Diagnostic Accuracy Studies.* Clin Chem, 2015. **61**(12): p. 1446-52.

35. Björnson, E., et al., *Lipoprotein(a) Is Markedly More Atherogenic Than LDL: An Apolipoprotein B-Based Genetic Analysis.* J Am Coll Cardiol, 2024. **83**(3): p. 385-395.

36. Helm, B.M., V. Huerta, and J.M. Clary, *Missed and Misdiagnosed: Precision Medicine Approaches to Screening for Familial Hypercholesterolemia and Lipoprotein(a).* JACC Adv, 2025. **5**(1): p. 102461.

37. Wand, H., et al., *Genetic testing preferences and intentions in patients with clinically diagnosed familial hypercholesterolemia.* J Genet Couns, 2020. **29**(6): p. 919-927.

38. Ibrahim, S., et al., *Enhanced identification of familial hypercholesterolemia using central laboratory algorithms.* Atherosclerosis, 2024. **393**: p. 117548.

39. Bhatia, H.S., et al., *Lipoprotein(a) Testing Trends in a Large Academic Health System in the United States.* J Am Heart Assoc, 2023. **12**(18): p. e031255.

40. Shah, N.P., et al., *Lipoprotein (a) Testing in Patients With Atherosclerotic Cardiovascular Disease in 5 Large US Health Systems.* J Am Heart Assoc, 2024. **13**(21): p. e035610.

41. Ellis, K.L., et al., *Familial combined hyperlipidemia and hyperlipoprotein(a) as phenotypic mimics of familial hypercholesterolemia: Frequencies, associations and predictions.* J Clin Lipidol, 2016. **10**(6): p. 1329-1337.e3.

42. Pepplinkhuizen, S., et al., *Electronic health records to facilitate continuous detection of familial hypercholesterolemia.* Atherosclerosis, 2020. **310**: p. 83-87.

43. Qureshi, N., et al., *Comparing the performance of the novel FAMCAT algorithms and established case-finding criteria for familial hypercholesterolaemia in primary care.* Open Heart, 2021. **8**(2).

44. Damgaard, D., et al., *The relationship of molecular genetic to clinical diagnosis of familial hypercholesterolemia in a Danish population.* Atherosclerosis, 2005. **180**(1): p. 155-60.

45. Civeira, F., et al., *Comparison of genetic versus clinical diagnosis in familial hypercholesterolemia.* Am J Cardiol, 2008. **102**(9): p. 1187-93, 1193.e1.

46. Schmieder, R.S., et al., *Clinical scores fail to sufficiently identify children with familial hypercholesterolaemia.* Eur J Prev Cardiol, 2026. **33**(3): p. 361-369.

47. Samadder, N.J., et al., *Exome Sequencing Enhances Screening for Familial Hypercholesterolemia Within a Multi-Site Healthcare System.* Circ Genom Precis Med, 2025: p. e005174.

48. Kronenberg, F., et al., *Lipoprotein(a) in atherosclerotic cardiovascular disease and aortic stenosis: a European Atherosclerosis Society consensus statement.* Eur Heart J, 2022. **43**(39): p. 3925-3946.

49. Alonso, R., et al., *Familial Hypercholesterolemia and Lipoprotein(a): Two Partners in Crime?* Curr Atheroscler Rep, 2022. **24**(6): p. 427-434.

50. Koschinsky, M.L., et al., *A focused update to the 2019 NLA scientific statement on use of lipoprotein(a) in clinical practice.* J Clin Lipidol, 2024. **18**(3): p. e308-e319.

51. Murray MF, E.J., Angrist M, Chan K, Uhlmann W, Doyle DL, and G.T. Fullerton SM, Hagenkord J, Imhof S, et al, *A proposed approach for implementing genomics-based screening programs for healthy adults.* National Academy of Medicine Persepectives 2018.
